# Supplementary material for: An exploration into CTEPH medications: Combining natural language processing, embedding learning, in vitro models, and real-world evidence for drug repurposing
Source: PLoS Comput Biol. 2024 Sep 12;20(9):e1012417. doi: 10.1371/journal.pcbi.1012417 (PMC11478854; doi:10.1371/journal.pcbi.1012417)
Supplement: S1 Fig — A) Query Phrase dataset: chronic thromboembolic pulmonary hypertension, B) Associated Disease dataset: venous thrombosis. Overview of number of terms per abstract (y-axis) within the special term categories "Drug Compound" and "Clinical Feature" (hue). Four filters have been added (x-axis): “All Terms” includes all terms within the term category. “Unique Terms” removes all duplications of a term within an abstract. The suffix "No Empty Abstract" removes all abstracts without a term within the term category. Log-scaling of the y-axis enhances visualization of low frequency term occurrences. Retrieval date: July 11, 2024. (PDF) [file pcbi.1012417.s001.pdf]

Literature Corpora Characterization

In this section, we provide graphical (Figure S1) and statistical (Table S1) characterizations of the respective literature corpora obtained for the Query Phrase: chronic thromboembolic pulmonary hypertension, and the Associated Disease: venous thrombosis (retrieval date: July 11, 2024). Terms in these two literature corpora are separated into term groups: “Drug Compound Terms” and “Clinical Feature Terms”.

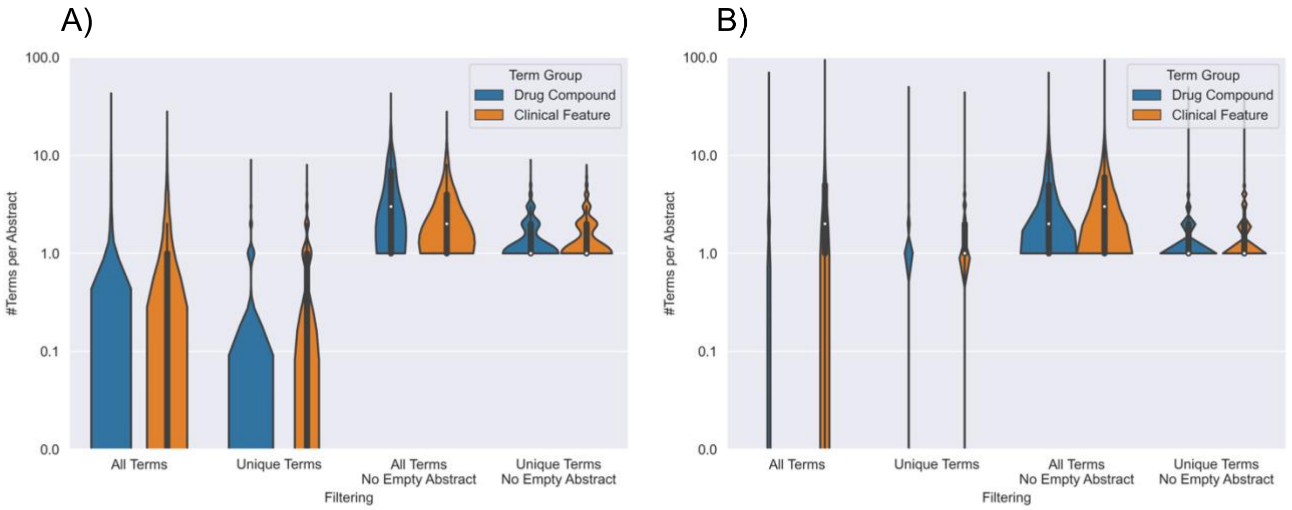

S1 Fig: Characterization of the Query Phrase and Associated Disease literature corpora. A) Query Phrase dataset: chronic thromboembolic pulmonary hypertension, B) Associated Disease dataset: venous thrombosis. Overview of number of terms per abstract (y-axis) within the special term categories "Drug Compound" and "Clinical Feature" (hue). Four filters have been added (x-axis): “All Terms” includes all terms within the term category. “Unique Terms” removes all duplications of a term within an abstract. The suffix "No Empty Abstract" removes all abstracts without a term within the term category. Log-scaling of the y-axis enhances visualization of low frequency term occurrences. Retrieval date: July 11, 2024.
