# Supplementary material for: An exploration into CTEPH medications: Combining natural language processing, embedding learning, in vitro models, and real-world evidence for drug repurposing
Source: PLoS Comput Biol. 2024 Sep 12;20(9):e1012417. doi: 10.1371/journal.pcbi.1012417 (PMC11478854; doi:10.1371/journal.pcbi.1012417)
Supplement: S1 Table — (PDF) [file pcbi.1012417.s002.pdf]

S1 Table: Statistical measures describing the composition of the literature corpora.

**Query Phrase corpus:** chronic thromboembolic pulmonary hypertension			
**Drug Compound Terms**			
3614 documents		532 documents (No Empty Abstract)	
Mean	Standard deviation	Mean	Standard deviation
0.7369 terms	2.5768	4.7384 terms	4.8721
0.2443 unique terms	0.6849	1.5712 unique terms	0.9653
**Clinical Feature Terms**			
3614 documents		1353 documents (No Empty Abstract)	
Mean	Standard deviation	Mean	Standard deviation
1.1597 terms	2.3367	3.0976 terms	2.9295
0.6475 unique terms	1.0904	1.7295 unique terms	1.1421
**Associated Disease corpus:** venous thrombosis			
**Drug Compound Terms**			
82267 documents		13672 documents (No Empty Abstract)	
Mean	Standard deviation	Mean	Standard deviation
0.6569 terms	2.3628	3.9527 terms	4.5349
0.2386 unique terms	0.6627	1.4359 unique terms	0.9611
**Clinical Feature Terms**			
82267 documents		67263 documents (No Empty Abstract)	
Mean	Standard deviation	Mean	Standard deviation
3.4081 terms	3.8306	4.1683 terms	3.8442
1.5300 unique terms	1.3332	1.8713 unique terms	1.2391

"No Empty Abstract": Abstracts with no term category terms are removed
"Terms": All term category terms within an abstract are included
"Unique Terms": Duplicate term category terms within an abstract are removed
