# Supplementary material for: An exploration into CTEPH medications: Combining natural language processing, embedding learning, in vitro models, and real-world evidence for drug repurposing
Source: PLoS Comput Biol. 2024 Sep 12;20(9):e1012417. doi: 10.1371/journal.pcbi.1012417 (PMC11478854; doi:10.1371/journal.pcbi.1012417)
Supplement: S2 Table — (PDF) [file pcbi.1012417.s003.pdf]

Special Terms: Clinical Feature Identifiers

To identify clinical features/terms within our acquired literature corpora, we compiled the following list of suffixes based on a general medical glossary (Table S2).

S2 Table: Compiled list of suffixes used to identify clinical features from the literature corpora.

SUFFIX	MEANING
-algia	Pertaining to pain
-astheno	Weakness
-blast	Immature cell
-cele	Pertaining to a tumor or swelling
-centesis	Pertaining to a procedure in which an organ or body cavity is punctured, often to drain excess fluid or obtain a sample for analysis
-cyte	Cell
-emia	Pertaining to the presence of a substance in the blood
-genic	Causing
-itis	Inflammation
-lysis	Decline, disintegration, or destruction
-megaly	Enlargement of
-oma	Tumor
-osis	Pertaining to a disease process
-pathy	Disease or a system for treating disease
-phagia	Pertaining to eating or swallowing
-phasia	Pertaining to speech
-phobia	Pertaining to an irrational fear
-plegia	Paralysis
-pnea	Pertaining to breathing
-ptosis	Drooping
-rrhage	Abnormal or excessive flow or discharge
-rrhagia	Abnormal or excessive flow or discharge
-rrhaphy	Suture of; repair of
-rrhea	Flow or discharge
-sis	A process, action, or condition
-trophic	Pertaining to nutrition
-uria	Pertaining to a substance in the urine or the condition so indicated
