# Supplementary material for: An exploration into CTEPH medications: Combining natural language processing, embedding learning, in vitro models, and real-world evidence for drug repurposing
Source: PLoS Comput Biol. 2024 Sep 12;20(9):e1012417. doi: 10.1371/journal.pcbi.1012417 (PMC11478854; doi:10.1371/journal.pcbi.1012417)
Supplement: S1 Text — (PDF) [file pcbi.1012417.s004.pdf]

S1 Text: Comparison with Alternative Methods: PrimeKG and DREAMwalk

In this section, we provide a comparison of our tool, PubDigest, with two other state-of-the-art knowledge graph approaches: PrimeKG and DREAMwalk. These comparisons offer insights into the robustness and applicability of our method, particularly for rare diseases such as Chronic Thromboembolic Pulmonary Hypertension (CTEPH).

PrimeKG (https://pubmed.ncbi.nlm.nih.gov/36732524/)

PrimeKG is a comprehensive knowledge graph that integrates various biomedical data sources, aiming to provide a holistic and multimodal view of diseases. Our attempt to use PrimeKG for CTEPH involved several steps:

1. **Installation**: We faced significant difficulties installing PrimeKG using both pip and conda on multiple devices running Windows and Linux. However, we were able to review their provided knowledge graph to identify relevant connections.
2. **Data Exploration**: Within the PrimeKG knowledge graph, we identified two drugs associated with CTEPH: "riociguat" (the current gold-standard treatment) and "terguride" (an older, discontinued drug). Additionally, the parent disease of CTEPH was identified as “pulmonary hypertension” and related disease phenotypes were identified, with the top five including: increased pulmonary vascular resistance, palpitations, deep venous thrombosis, reduced vital capacity, and exertional dyspnea. Expected drugs that are known to be associated with CTEPH such as bosentan, sildenafil, and iloprost were notably not identified.
3. **Limitations**: Due to installation issues, we were unable to fully test PrimeKG, although a complete knowledge graph was available for download.

Furthermore, despite PrimeKG's impressive incorporation of curated primary data resources, we noted that some relevant CTEPH drugs were not listed as associated with CTEPH in their provided knowledge graph (bosentan/sildenafil/iloprost), demonstrating lacking node connections for rare diseases like CTEPH may be likely. Lastly, PrimeKG's predictive capabilities are limited, as while phenotypes are suggested, implementation of edge prediction on these phenotypes is not straightforward.

The full PrimeKG output for nodes connected to CTEPH is available in our Dataverse repository. https://doi.org/10.7910/DVN/TWZAGW

DREAMwalk (https://pubmed.ncbi.nlm.nih.gov/37322032/)

PrimeKG is a knowledge graph tool designed to facilitate biomedical research and drug repurposing, and uses a semantic information-guided random walk to map drugs and diseases in a unified embedding space. Our exploration of DREAMwalk's functionality identified the following:

1. **Installation**: The installation of DREAMwalk was error-free and straightforward. However, defining inputs presented challenges, requiring pre-curation of network files, semantic hierarchies, and node type labels using additional external tools and databases.
2. **Data Exploration**: While we identified idiopathic pulmonary artery hypertension (another sub-type of pulmonary hypertension) within DREAMwalk's provided knowledge graph, CTEPH was notably not contained. This absence restricted our ability to investigate CTEPH using DREAMwalk.
3. **Limitations:** The limited coverage of DREAMwalk underscores a broader issue: knowledge graphs often inadequately cover rare diseases like CTEPH.

This highlights the necessity for tools like PubDigest that crawl and integrate biomedical literature to ensure comprehensive drug repurposing. Furthermore, the necessity of pre-curated and thorough input files generated externally complicates the general usability of this tool for a broader userbase and creates a reliance on external databases and tools to be maintained.

Summary of Findings

Both PrimeKG and DREAMwalk fall short in addressing rare diseases easily and comprehensively. PubDigest fills this gap by offering an alternative approach that integrates biomedical literature to develop drug repurposing predictions for any desired input term that exists in the literature corpus.
