# Supplementary material for: An exploration into CTEPH medications: Combining natural language processing, embedding learning, in vitro models, and real-world evidence for drug repurposing
Source: PLoS Comput Biol. 2024 Sep 12;20(9):e1012417. doi: 10.1371/journal.pcbi.1012417 (PMC11478854; doi:10.1371/journal.pcbi.1012417)
Supplement: Attachment — Submitted filename: Rebuttal Textcrawler_Comments Reviewers_Szulcek.pdf [file pcbi.1012417.s005.pdf]

Rebuttal PCOMPBIOL-D-23-01945

An Exploration into CTEPH Medications: Combining Natural Language Processing, Embedding Learning, *In Vitro* Models, and Real-World Evidence for Drug Repurposing

Reviewer 1

We thank the reviewer for his favorable comments and for recognizing the reasoning of our manuscript. We appreciate the acknowledgment that our focus is on creating a comprehensive pipeline for curating candidate drugs for *in silico* and *in vitro* evaluations in a specific clinical context for repurposing.

We are pleased that the reviewer found our background summary and methods clear and that he appreciates our inclusion of follow-up analyses such as *in vitro* thrombogenicity and drug safety assessments. We also value the recognition of our efficient pathway to drug repurposing.

We addressed the minor comments raised to further enhance our manuscript.

Thank you once again for your constructive review.

Q1: In the third paragraph of the results, starting on line 187, the authors indicate that "riociguat enhances cardiopulmonary hemodynamics by stimulating soluble guanylate cyclase (sGC) activity that ranks fourth in our analysis". The fact that sGC activity ranks fourth in the analysis is difficult to interpret without further context; this should be clarified.

A1: While most of the listed compounds are drugs, the term "guanylate" appears due to the antihypertensive prefix "guan-" used in drug names according to USAN. This inclusion serves as a nice sanity check, reflecting riociguat's mechanism, which is often described as "enhances cardiopulmonary hemodynamics by stimulating soluble guanylate cyclase (sGC) activity." Both terms show similar TF-IDF profiles, with riociguat ranking first and guanylate fourth in overall mentions. This prefix, infix, and suffix approach for drug identification is essential to comprehensively include all relevant drug compounds, as other methods like ChEMBL database querying resulted in unacceptable exclusions. This also explains the presence of

terms such as (guan-)ine and (trop-)onin. We have clarified these points in the manuscript. For further reference, please see our response to question 4 of Reviewer 2.

Q2: In the second paragraph of the section "Word2Vec Embedding Reveals Potential Drug Candidates for CTEPH", on p. 9, Word2vec should be cited when it is first mentioned (line 218).

A2: Thank you for pointing this out, as the order of references changed because of the format required by PLOS, having the methods at the end of the manuscript. We have now fixed this by adding the citation to the first mention of Word2Vec. The same has been done to TF-IDF, which is now cited at first mention.

Q3: Later in this same paragraph, the authors indicate that "Manual curation was needed, as the model doesn't distinguish positive from negative associations/sentiments (as in inducing or inhibiting thrombosis)." If there were additional reasons that manual curation was needed these should also be mentioned.

A3: Specifically, the model generated a list of indirect term associations but is not capable of correlating these with known drug actions. It did not contextualize these drugs as inducers or inhibitors of VT or as pro- or anti-thrombotic agents. Hence, manual curation was necessary to assess the known effects of the identified drugs and to ensure the relevance and accuracy of the drug associations identified. This was the sole reason for manual curation. We have clarified this in the text by stating that "No additional manual curation was undertaken on the output" and have revised the paragraph for clarity. In general, a couple of manual inputs are required by the user, which we specified in our reply to question 3 of Reviewer 2 and updated the manuscript accordingly.

Q4: In Figure 1B, in the "Module 1.0" panel, the heading under "Process Bibliometric Data" reads "Influential Authors by Country and City". However, there is no mention of an analysis related to influential authors in the manuscript. This should either be addressed in the manuscript or removed.

A4: Our tool can generate a citation analysis and identify the most influential authors by country. However, this functionality was not relevant for this manuscript, so we removed it from the flow chart. See below an example output from our tool. In a similar fashion, we removed "a) Abbreviations" and the associated description from the text, since it was not used. See also the reply to Q12 of reviewer 2 for more information.

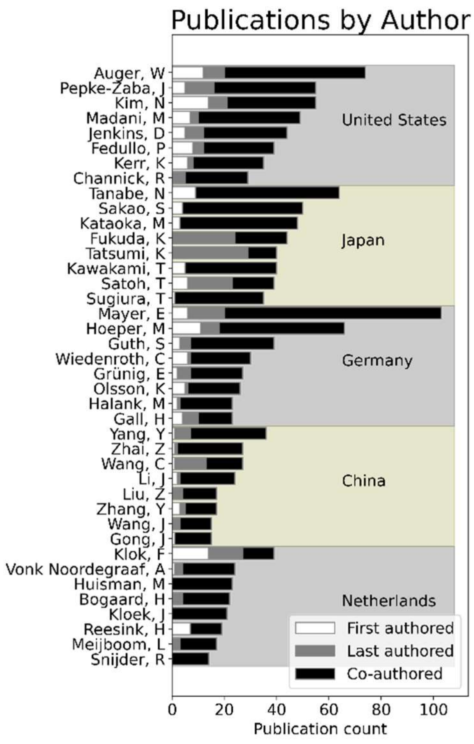

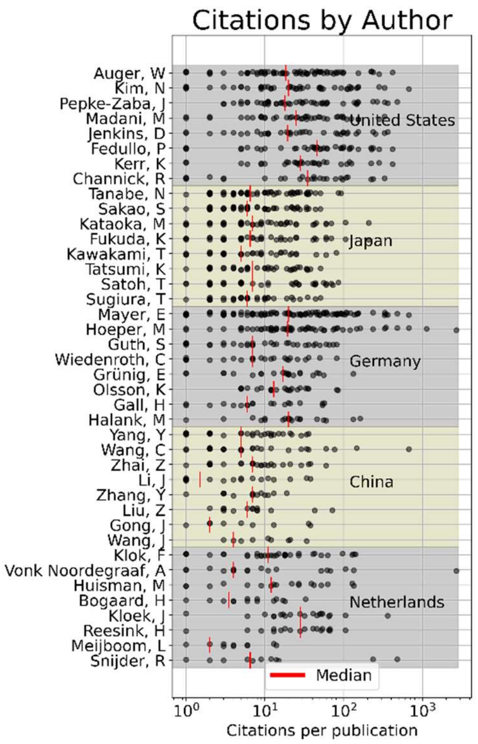

Q5: In Figure 2A (the word cloud) the individual words appear to vary with respect not only to size, but also color and opacity. If the variance of these visual properties is meant to convey information, the legend should be updated to reflect this.

A5: The grayscale value and opacity of the word cloud do not convey any specific information but serve as a visual aid to distinguish between individual terms. We have added a sentence to the description of Figure 2 to clarify this: “Color and opacity do not convey information but enhance readability.”

Reviewer 2

We thank the reviewer for the thorough and insightful comments on our manuscript. We appreciate the recognition of our manuscript as an intriguing combination of computational approaches, encompassing automatic literature research, toxicity and biological assessment of drug action, as well as survival and risk analysis using clinical data.

We also appreciate the feedback on the areas needing further clarity and detail. This constructive critique guided us in enhancing the presentation of our results, particularly the intermediate findings. We address the need for a more detailed comparison with state-of-the-art literature mining approaches and a clearer explanation of the manual curation steps involved.

Thank you again for your constructive suggestions. We incorporated these improvements to strengthen the manuscript and better convey the robustness and applicability of our pipeline.

Q1: Neither the data repo (claiming to provide intermediate results) nor the software repo are available under the provided links!

A1: Thank you for pointing this out! Mistake on our side. These repositories are now public under the provided links.

Q2: The work fails to cite newer knowledge graph approaches such as PrimeKG [https://www.nature.com/articles/s41597-023-01960-3] (there are only two citations from 2015 and 2020). Also, what would be the outcome of investigating CTEPH and related drugs with these databases? Is it possible or not possible? This would be an important orthogonal check to determine the benefit of the proposed tool vs. the state-of-the-art.

A2: We have now cited PrimeKG and DREAMwalk in the introduction and drew comparisons in the discussion of the manuscript.

Investigating CTEPH with these two tools was challenging. We attempted to investigate CTEPH and related drugs with PrimeKG but were unable to install it both with pip and conda on both Windows and Linux, trying multiple devices. However, by looking into their provided knowledge graph we identified CTEPH and all its edges. In total, we found two drugs, “riociguat”, the current gold-standard treatment for CTEPH, as well as a drug called “terguride”, an old and discontinued drug from the 1980s that acts on a pathway that was initially thought to cause another sub-type of pulmonary hypertension, which was not confirmed empirically. Consequently, the parent disease of CTEPH was identified: “pulmonary

hypertension”. The full list for PrimeKG on CTEPH is uploaded to the Dataverse repository. Unfortunately, due to the failed installs, we could not test the link prediction capabilities of this tool.

DREAMwalk was easy to install, although defining inputs proved challenging, thus we took a closer look into their knowledge graph files. While (idiopathic) pulmonary artery hypertension could be identified, CTEPH was not contained in the list of diseases, restricting its ability to be investigated.

These two alternative approaches showed that understudied/rare diseases, such as CTEPH, are poorly covered in knowledge graphs, if at all. Based on general clinical treatment strategies and published research, we expected more drugs to be associated with CTEPH, such as bosentan, sildenafil, and iloprost. Usability/ease of use was a second hurdle with these two tools, as numerous basic researchers in our research group, with less bioinformatic experience, were unable to successfully apply PrimeKG and DREAMwalk.

We have nevertheless considered this question deeply and understand that the original submission may have implied the tool is a strict improvement on the existing software options. This was not our intention, and we have revised the manuscript’s introduction to emphasize that we are not attempting to outperform other tools, but rather to apply an alternate approach integrating biomedical literature and support its application by our real-world, tox prediction, and *in vitro* validation pipeline to judge the efficacy of the compound both for its proposed mechanism and clinical suitability.

Q3: Throughout, manual interventions need to be clearly marked, and their result (outcome of the tool – steps for manual curation and why – result after manual curation) need to be provided to the reader, and potentially the robustness with respect to the choices made need to be assessed. If space becomes an issue, this can well be provided in the Supplementary, but these are important steps of a decision process that need to be conducted when wanting to use this tool for a different case.

Examples for this are

(a) How many and which words did you have of each category of CTEPH abstract (clinical, abbreviations, drug compounds)? How many words were used for the word cloud?

(b) For the n-gram analysis: What were the full results for the analysis output from the pipeline? And what does “delivered the most explicit results” mean in this context? What are detailed intermediate results that are gotten from the pipeline for n-grams and how were they handled?

What were the results for n-grams with n>2? Why did you choose the top 10 clinical terms for the n-gram analysis? What would happen using the top 12 or 15 or 30?

(c) Word2vec: What does “narrow the focus to drug terms” mean? Was inference only done on drug terms? Or was also the pre-training affected?

A3: **User required inputs:** There are only three instances of manual interventions necessary, which are simple query inputs. As an overview, manual inputs are as follows:

1. Define a “Query Phrase” (here “chronic thromboembolic pulmonary hypertension”): This is pointed out in the manuscript in the Methods, calling it a user-defined “Query Phrase”.
2. Select an “Associated Disease”, can be selected from the list provided (exploratory approach) or set by the user independently (targeted approach) (here “venous thrombosis”): This is mentioned in the word2vec methods section of the manuscript, “From the generated list of disease terms, we manually chose the “Associated Disease” ("venous thrombosis") as a targeted approach to this study.”.
3. Define a “Prediction Term”, which is used to search in the word2vec embedding layer for direct and indirect drug compound term associations (here “CTEPH”): This is pointed out in the manuscript in the methods section Word2Vec Term Association, “The final part of this module involves searching the embedding layer for the “Prediction Term”, here “CTEPH”.”.

The workflow of our tool runs without any interventions if all three terms are set.

Optional customization: Overall, PubDigest is highly customizable, such that terms can be excluded from special term section, different numbers of terms can be plotted, Query Phrases and Associated Disease Terms can reflect advanced PubMed queries, n-gram numbers can be adapted to the respective needs, multiple Alternative Disease Terms can be run with a single execution, TF-IDF intervals can be predefined, color schemes can be adapted, execution of single steps or a subset of steps can be applied. All these parameters are adjustable via configuration files. However, we do not recommend this to the user.

Specific examples:

(a)

- WordCloud: All terms were used to generate the word-cloud. Only the top 100 are shown to keep the image clean.
- Drug Compounds:
    i. No. total Terms per Document: 2597 Documents; Mean #Terms: 0.81; standard deviation #Terms: 2.7
    ii. No. unique Terms per Document: 2597 Documents; Mean #Terms: 0.26; standard deviation #Terms: 0.7

iii. No. total Terms per Document – Documents with no entry dropped: 431 Documents; Mean #Terms: 4.86; standard deviation #Terms: 4.94
iv. No. unique Terms per Document – Documents with no entry dropped: 431 Documents; Mean #Terms: 1.58; standard deviation #Terms: 0.93
v. Figure of Distribution are added to Dataverse Repository (Drug_Compounds_Term_length.png)
vi. List of all Terms is added to the Dataverse Repository (Special_Terms.csv)

- Disease Terms:
  i. No. total Terms per Document: 2597 Documents; Mean #Terms: 1.18; standard deviation #Terms: 2.36
  ii. No. unique Terms per Document: 2597 Documents; Mean #Terms: 0.67; standard deviation #Terms: 1.1
  iii. No. total Terms per Document – Documents with no entry dropped: 431 Documents; Mean #Terms: 3.09; standard deviation #Terms: 2.94
  iv. No. unique Terms per Document – Documents with no entry dropped: 431 Documents; Mean #Terms: 1.75; standard deviation #Terms: 1.13
  v. Figure of Distribution are added to Dataverse Repository (Clinical_Terms_Term_length.png)
  vi. List of all Terms is added to the Dataverse Repository (Special_Terms.csv)
- Abbreviations have been removed from this manuscript, as discussed in our responses to Reviewer 1, Question 4.

(b) n-grams analysis: We added these and other program output steps to the Dataverse repository. The respective files are listed below. These files are generated within the program and are the basis for the shown figures. We excluded the sentence: “returned the most explicit results”, since it apparently resulted in confusion about the actual approach. The remark was intended to comment on the dominance of 2-grams measured by count of occurrences. The approach utilizes n-grams of size 2-5. The selection of top 10 “Clinical Features” has been chosen by us without a given metric, to exclude noise. To showcase alternative top X Clinical Features’ influence on the results, we generated a table including all Clinical Features identified. This resulted in a very noisy output, mainly driven by term “analysis”, which is defined as a clinical term through its suffix, but through its ubiquitous usage attained a low TF-IDF score. Through iterative processing of the data, exclusions of terms have been defined such as “analysis”, “be”, and others. Excluding this noise improves readability of results, but also requires additional, optional inputs. Increasing the top X Clinical Features, can be

helpful in find additional diseases such as sleep apnea (42 mentions), systemic sclerosis (25 mentions), nocturnal hypoxemia (25 mentions) fibrosing mediastinitis (20 mentions), rheumatoid arthritis (18) and others. The full results of this extension are added to the Dataverse repository, along with the here published list of top 10 Clinical Features.

- Dataverse files:
  - Crawling Dataset CTEPH: "CTEPH_Dataset.xlsx"
  - Crawling Dataset VT: "VT_Dataset.xlsx"
  - KG CTEPH Output: "PrimeKG_connected_nodes_to_CTEPH.csv"
  - Disease Term Counts (all Clinical Features): "Disease_names_counts_ALL_clinical_features.csv"
  - Disease Term Counts (top 10 Clinical Features): "Disease_names_counts_top10_clinical_features.csv"
  - Drug Compound term dataset: "TF_IDF_Drugs.csv"
  - Clinical term dataset: "TF_IDF_Clinical_Features.csv"
  - Drug compound ranking CTEPH: "Embedding_Layer_Similarity_Annotated_Skip-gram.csv"
  - Manual curation of terms by their thrombotic effects: "Thrombotic Annotation.csv"

(c) "Narrow the focus to drug terms" means, the model got trained on all terms, and we dropped all non-drug terms for our drug term analysis. We have revised this sentence for clarity as follows: "We then depopulated the embedding space to only include drug compound terms and the "Prediction Term", CTEPH. Subsequently, we searched for the most similar drug compound terms to the "Prediction Term" using the cosine similarity metrics (Figure 4)."

Q4: Entity recognition was mentioned as a problem of knowledge-based approaches. But also in their approach, entity recognition plays a role. How was ensured that their entities were recognized well? I also see that in the word-cloud, e.g., anti-cardiolipin as well as anticardiolipin (with and without hyphen) appear as separate entities, suggesting a lack of term normalization. Plus, there seems to be "noncardiac" and "chemoattractant" in the list of drug compounds – both suggesting that entity recognition might not have worked well here. Please introduce steps to control for this, or at least comment on why this is not a problem in your case, and provide and describe intermediate output for the reader.

A4: In addition to our response to Q1 of Reviewer 1, we address entity recognition and term normalization in our approach. The curation of drug compounds relies on the United States Adopted Names (USAN) approved stems, which provide common stems based on established

chemical and pharmacologic parameters, guiding the naming of new drug compounds within related series. Due to the nature of pharmacological terminology, compound prefixes and suffixes often overlap with associated phenotypes, diseases, or molecules, leading to occasional inclusion of non-drug terms such as “noncardiac” and “chemoattractant.” We consider this a necessary level of noise to capture all potentially relevant drug compounds.

We previously attempted to use the ChEMBL database for compound curation but encountered significant gaps where expected relevant compounds were missing. By adopting a “noisier” but more comprehensive approach based on USAN stems, we increased the number of identified drug compounds in the CTEPH case study from 110 with ChEMBL to 197 with USAN. This approach yielded higher sensitivity in exchange for specificity, which we deemed more important.

We have elaborated on this methodology in the Methods section to clarify our approach. The list of USAN stems utilized is cited in the text and is freely available for download via the link provided on the GitHub page for the tool. While we recognize minor issues in term normalization — variations in hyphenation, alternate spellings, and misspellings — we have not yet developed an automated solution to address these inconsistencies. Despite these challenges, our approach ensures a comprehensive capture of relevant terms, which we believe is critical for the robustness of our analysis.

Q5: TF-IDF: Why this and not some other citation relevance metric, e.g., page rank, in a citation graph? What are the benefits? Is it performing better? Or is it particularly fast? This is a point where control experiments and comparisons with other approaches are missing. And what is the benefit from interval-normalized TF-IDF, except for more resolved figures? The development is not considered here at all for advancing in the pipeline, as far as I can see.

A5: TF-IDF: We chose TF-IDF for several compelling reasons. First, its equation is simple and easily understandable, making it accessible to our interdisciplinary team, including biologists and medical staff who may not have extensive computational backgrounds. This simplicity facilitates effective communication and collaboration across different expertise areas. Additionally, TF-IDF is computationally efficient, allowing us to quickly process and analyze large datasets such as PubMed abstracts without requiring extensive computational resources. We also could easily adapt TF-IDF to our specific needs by introducing interval-weighted TF-IDF, which accounts for the temporal aspects of term relevance and provides a more nuanced understanding of literature trends over time. This customization was crucial for our study's dynamic and evolving medical research context. Furthermore, our use of TF-IDF yielded results that aligned with our expectations based on domain knowledge of our medical

colleagues, which validated its effectiveness for our purposes. Given this satisfactory performance, we prioritized advancing our study rather than benchmarking against other term ranking methods. Finally, benchmarking different citation relevance metrics can be challenging due to the lack of a definitive ground truth. While algorithms such as TextRank (an adaptation of the PageRank algorithm) or BM25 (an extension of TF-IDF) could potentially be applicable, determining which metric is objectively better it is not straightforward. In our case, TF-IDF provided a robust and reliable metric based on our domain expertise, negating the immediate need to explore alternative metrics.

Interval-weighted TF-IDF: The introduction of interval-weighted TF-IDF, while not directly aiding the automated pipeline, provides significant benefits in offering a temporally resolved view of the data. This method allows users to distinguish between established features and novel developments in the field over specific time intervals. By examining how the relevance of terms changes over time, researchers can gain insights into emerging trends, shifts in research focus, and the evolution of scientific discourse. This temporal resolution is crucial for understanding the dynamic nature of medical research and can guide further investigations and strategic decisions. Overall, we feel that the interval-weighted TF-IDF contributes to a deeper and more contextual understanding of the literature, and it makes beautiful graphs, too.

Q6: Venous thrombosis is quite obviously associated with CETPH. What is the benefit of using venous thrombosis as secondary disease literature corpus instead of, e.g. “(acute) pulmonary embolism” that would have been the most natural choice from clinical experience (as written in the discussion)? Describing this properly would enhance the reasoning on the usefulness of the methods (as in: what do you get by the method that you would not have gotten from the start using a less work intensive than your method). Also, what would happen if using the second best clinical association after venous/vein thrombosis as a second literature corpus?
A6: Choosing an associated disease for the secondary literature corpus requires domain knowledge, which we aimed to minimize as a prerequisite for using our tool. Venous thrombosis (VT, same as vein thrombosis) is often a precondition for acute pulmonary embolism (PE), with 50% of untreated VT cases developing into PE. Therefore, the VT literature inherently includes substantial content related to PE, allowing us to demonstrate that deep domain knowledge is not essential to obtain reasonable inputs using our tool. Additionally, the VT corpus is larger, offering more extensive data and potential connections. This is important as antithrombotic treatments for VT and acute PE are generally the same.

Using another disease corpus, such as acute pulmonary embolism, would likely yield different results, potentially offering valuable insights as well. However, our focus was on selecting the top-ranked term, for this case study. This approach ensured a robust and comprehensive analysis, illustrating the tool's capability without extensive manual input. The flexibility of our method allows users to manually input other associated diseases for further exploration, which we discussed previously.

Q7: What is the full list of intermediate drug results, both direct and indirect? Without this list and a proper description of what exactly was done for manual curation, the extent of manual curation required to come up with the final list of drugs is not possibly judged. However, this is a crucial step in drug repurposing.

A7: The annotated table and table used for annotation is added to the Dataverse Repository (Embedding_Layer_Similarity_Annotated_Skip-gram.csv; Thrombotic_Annotation.csv). Curating this data is done by searching for direct term associations, terms already present in the query dataset (Query Phrase: “Chronic Thromboembolic Pulmonary Hypertension”), or more precisely by co-occurrence with CTEPH in an abstract or title. All other terms are indirect term associations. To further analyze the data, manual curation was necessary, as we explained in detail in the reply to Q3 of reviewer 1. The annotation process is now described in detail in the results section of the manuscript: “The latter underwent manual curation, cross-referencing drug databases and literature (www.drugs.com, www.mmi.de, www.pubmed.ncbi.nlm.nih.gov) to discern anti-thrombotic from pro-thrombotic properties”.

Q8: The transition from benzarone to the eventually examined drug amiodarone is unclear. I think there could be many other derivates with similar molecular structures. Why were these not chosen? This is important for being able to transfer this analysis to other cases.

A8: A structural similarity search identified several alternative compounds within the same drug family, which were then screened for their toxicological profiles. Amiodarone was selected for further evaluation due to its manageable toxicity and established history of clinical use. The transition from benzarone to amiodarone was based on retaining the benzarone core structure while prioritizing compounds with clinical acceptance and manageable toxicity.

The selection process can be summarized as follows:

1. Identifying and maintaining the structure essential for biological activity.
2. Screening for similar compounds and considering metabolic retention.
3. Filtering for well-documented compounds.
4. Selecting compounds based on toxicity and clinical acceptance.

We have added a detailed description of amiodarone selection to the results section. While alternative approaches could automatically output a list of structurally preserved compounds (*e.g.*, via PubChem), these methods require extensive automatic or manual curation and filtering. For example, a structural search for benzarone yielded 3,727 potential molecules retaining the benzarone substructure. A quick literature scan can sometimes be more productive and accurate than automated approaches for curating a list of actively used and well-described molecules in the medical space, rather than providing a broad overview of all structurally similar molecules. This process exemplifies the practical challenges that need to be addressed in drug repurposing efforts.

Q9: It remains unclear in how far and for which steps the cited approach of Tshitoyan et al. is followed. The relations between the contributions here to the existing work need to be made clearer.

A9: The approach from Tshitoyan et al represents the fundamental idea we applied to our objective of drug compound prediction from scientific literature. To adapt this idea from the material sciences to the medical field, we established special terms that reflect the biomedical domain, such as drug compounds, clinical features, and disease names. The second, even more important distinguishing factor is the corpus used to construct the term embedding. While Tshitoyan et al used all abstracts they could find in their respective field, we joined the two disease specific datasets from a peer-reviewed data source and integrated them to create the term embedding. Similarities are the chosen model (word2vec), the similarity metric (cosine similarity), and the definition of direct and indirect term associations. We adapted the text in the manuscript accordingly (see Material & Methods – Word2Vec Term Association).

Q10: The abbr. TF-IDF is not introduced in the main text, only in the abstract, and it is not introduced in the results at all (only in the methods that come last in the document)

A10: Reordering the text for PLOS unfortunately resulted in the abbreviation and reference to be pushed to the back of the main text. We have corrected this formatting issue, introducing the abbreviation with the full name on the first appearance in the main text. Furthermore, the reference has been moved accordingly.

Q11: Fig. 1 is cited very last in the main text

A11: Thank you for pointing this out. Like before, this is a result of shifting the methods below the results. We have now introduced Fig 1 in the first paragraph of the results.

Q12: In the pipeline (Fig. 1), there is a step on “finding influential authors and cities” - What is the benefit of influential authors in the field? Is the literature list extended by this? This step is not described nor mentioned at all in the manuscript.

A12: The tool can generate a citation analysis and identify the most influential authors per country. This capability taken together with the interval weighted TF-IDF metric can identify trends in research. However, this functionality was not relevant to this manuscript, wherefore, we removed it from the flow chart. A possible, and very simple advantage of this functionality would be the detection of knowledge centers, where a disease is studied and where to find experts with whom to collaborate. See also the reply to Q4 of reviewer 1 for an example output.

Q13: It could be interesting for a reader which clinical studies/data resources could be used if they want to apply the approach to other cases.

A13: The usage of our tool is currently restricted to the PubMed Database. Including other resources such as clinical trial registries in text form can be facilitated in future development.

Q14: What about examining derivatives or similar drugs for the first-scoring drug, caplacizumab? Why were these not scrutinized? Also, without biological experiments, it would be very instructive for the reader to investigate the clinical impact of this drug according to the large clinical cohorts.

A14: Certainly, caplacizumab is a super interesting candidate! Unfortunately, as mentioned in the results section and discussion, we were unable to gain access to caplacizumab. Simply put, the manufacturer didn’t want to give us free samples and the drug is very expensive. So, we were forced to move to the second-scoring drug, benzarone.

Furthermore, caplacizumab is a nanobody, wherefore derivatives of caplacizumab are not available or existent. Toxicity screening of nanobodies is also not possible given our available tools, and caplacizumab is not contained in the TriNetX real-world data base restricting *in silico* evaluations. We have clarified these reasons for not performing such evaluations of caplacizumab in the methods (not in TriNetX), and discussion (not in drug toxicity database, nor TriNetX). We really hope that someone picks this finding up and tests caplacizumab in the CTEPH context.
