# Supplementary material for: An exploration into CTEPH medications: Combining natural language processing, embedding learning, in vitro models, and real-world evidence for drug repurposing
Source: PLoS Comput Biol. 2024 Sep 12;20(9):e1012417. doi: 10.1371/journal.pcbi.1012417 (PMC11478854; doi:10.1371/journal.pcbi.1012417)
Supplement: Attachment — Submitted filename: 240709_PubDigest_Revision1_comments.pdf [file pcbi.1012417.s006.pdf]

Response to the rebuttal R1, "An Exploration into CTEPH Medications: Combining Natural Language Processing, Embedding Learning, In Vitro Models, and Real-World Evidence for Drug Repurposing"

The authors have addressed the majority of my comments very well, thank you! However, some of the details they provide in the rebuttal should be findable also within the manuscript because they are beneficial for the reader and for making their data reproducible and FAIR. Therefore, I strongly recommend adding some further information and clarification into figure captions, methods and a newly to provide Supplementary Methods File with strongly required descriptions, as well as a missing dataset. Details are provided in the following.

My Q1 and Q3 - Datasets and their descriptions
The functioning Dataverse and github links make reproducibility a lot better, but it is not sufficient yet. You most definitely need a detailed dataset description - in the Dataverse Repo, it is not sufficiently resolved what is what. For that, a page (or two) in a Supplementary Methods File would be most suitable; and you should refer to this description when introducing the Dataverse Repo html address in the data availability statement. I suggest to use your Dataset list that you started off in response A3, correct for the shortcomings for the n-gram dataset as described below, and include descriptions for the missing 6 datasets (as you describe only 9 of 15 provided datasets). Please refer for each dataset to the Figure where the data was used (if applicable), this makes it a lot easier for the reader to follow and identify relevant datasets. Please also add a dataset that was used for the wordcloud in Fig. 2A, as these word frequencies are currently missing. Please also include your word occurrence characterizations from the rebuttal letter A3 (as a separate section) into the Supplementary Methods File that you could refer to in Fig. 2.

My Q3 - n-gram analysis and Fig. 3
Please comment in the caption of Fig. 3 that you only show the "X" n-grams with highest frequency, and that these happen to be only 2-grams, but that there are some 3-grams with frequency >6.
The files belonging to the n-gram analysis are not named such that one can make the link, e.g., "Disease_names_counts_ALL_clinical_features.csv" does not contain any hint on n-grams. Please alter the meta-description in the Dataverse, if possible, but at least the description of that dataset in the dataset list that I mentioned above. In addition, it appears that your results are truncated at a frequency of 6. Please describe this in the n-gram Methods part if you did this on purpose, and definitely also when describing the dataset.

My Q7 - Word2Vec-based similarity analysis and Fig. 4
Please add the following information into the caption of Fig. 4: the numbers of drugs with direct CTEPH associations, the numbers of drugs with indirect associations and positive or negative thrombotic effects, and mention that you only show the Top X (?) results. These characterizations are very valuable for the reader to get the full output data picture from your tool and truthfully reveals the fact that the list of potential drugs you obtain is quite long. However, as it is now, this remains highly hidden as it can only be derived by processing

some file from your Dataverse Repo. However, it should be very easy for you to derive and add.

My Q2 – Alternative methods PrimeKG and Dreamwalk
Great that you checked these approaches with CTEPH! Actually, your description in the rebuttal letter is much more convincing than your extremely shortened formulation in the discussion. I think they would be very valuable for the readers. Please include them into a Supplementary Methods section, and refer to this description in the discussion (instead of the link to the Dataverse that is a _lot_ less instructive without the interpreting guidance here).

Minor comment: The word "PubDigest" appears only exactly once (except for the running title), in line 353. I recommend to introduce somewhere explicitly what you mean with it.
