# Supplementary material for: An exploration into CTEPH medications: Combining natural language processing, embedding learning, in vitro models, and real-world evidence for drug repurposing
Source: PLoS Comput Biol. 2024 Sep 12;20(9):e1012417. doi: 10.1371/journal.pcbi.1012417 (PMC11478854; doi:10.1371/journal.pcbi.1012417)
Supplement: Attachment — Submitted filename: Rebuttal_PubDigest_R2.pdf [file pcbi.1012417.s007.pdf]

Response to the rebuttal R2, "An Exploration into CTEPH Medications: Combining Natural Language Processing, Embedding Learning, *In Vitro* Models, and Real-World Evidence for Drug Repurposing" (PCOMPBIOL-D-23-01945R1)

Thank you for your additional suggestions and comments! In addition to addressing your comments, we have also reprocessed all data to ensure they are up-to-date, subsequently updating Figures 1 to 4 (now Figures 2 to 5) and the graphical abstract (now Figure 1) to reflect the most recent information, given that the publication process has already spanned over seven months. Our findings remain consistent and reaffirm the usefulness and applicability of our tool, PubDigest. The changes have been tracked in the respective files uploaded to the Dataverse repository. Overall, our updates in this version are summarized as follows (note: no core conclusions have changed):

- Introduced and referenced the tool as PubDigest throughout the manuscript rather than "our tool"
- Numerous general grammatical and linguistic changes for clarity and readability
- Reprocessed the data (new retrieval date: 11 July 2024), resulting in changes in the # of abstracts per literature corpus, the total # of drugs identified in the reduced embedding space, disease term counts of occurrences, and the TF-IDF scores/rankings. <u>No top-ranked or influential changes occurred.</u> The exact changes are tracked in new Dataverse files named with the prefix "Changes_" and suffix "_Update"
- Annotated all Dataverse files and wrote a text file (available in Dataverse repository) called "Dataset_description.txt" that describes the datasets and their associations with the manuscript figures and methods
- Referenced "Dataset_description.txt" and Dataverse annotations in "Availability of data and methods" section
- Corrected that *all* clinical features were used to identify n-grams, rather than the top 10
- Clarified n-grams were mostly n=2, but included n-grams of n = [2,3,4,5] and were at a frequency > 5
- Mentioned in Figure 3 (now Fig. 4) caption how n-grams were restricted, and that the top 20 disease terms are shown
- Described in Figure 3 (now Fig. 4) caption that word cloud font sizes are derived from the drug's corpus-wide term frequency
- Added new Figure S1 and new Table S1 to characterize literature corpora datasets graphically and statistically, and referenced them in the manuscript

- Added new Supplementary Information section describing our attempts to apply PrimeKG and DREAMwalk for CTEPH, and referenced it in the Discussion
- Mentioned # of VT abstracts collected in text
- Further clarified and elaborated on how manual curation of pro- and anti-thrombotic drug compounds was performed (in both Methods and Results)
- Described in Results and Fig. 4 (now Fig. 5) caption how many total drugs were identified in the reduced embedding space and how many drugs were in each category (direct, indirect, pro-, anti-thrombotic)
- Mentioned in Fig. 4 (now Fig. 5) caption that the top 10 (direct association) or top 6 (indirect association pro- or anti-thrombotic) drug compounds are shown
- Increased number of direct association drug compounds shown in Fig. 4 (now Fig. 5) from 6 to 10 for additional information and to make it more comparable to the # of indirect association drug compounds that are shown also in Fig. 4 (now Fig. 5)
- Added the prefix “corpus-wide” or “interval-weighted” to all relevant TF-IDF mentions in text to clarify which method was used in each instance
- Minor methodology clarifications of the whole blood flow assay (Experimental *In Vitro* Validation) for accuracy and comprehensiveness
- Re-numbered figures to include Graphical Abstract as Fig. 1, shifting the rest of the figures +1

Reviewer: The authors have addressed the majority of my comments very well, thank you! However, some of the details they provide in the rebuttal should be findable also within the manuscript because they are beneficial for the reader and for making their data reproducible and FAIR. Therefore, I strongly recommend adding some further information and clarification into figure captions, methods and a newly to provide Supplementary Methods File with strongly required descriptions, as well as a missing dataset. Details are provided in the following.

My Q1 and Q3 - Datasets and their descriptions

The functioning Dataverse and github links make reproducibility a lot better, but it is not sufficient yet. You most definitely need a detailed dataset description - in the Dataverse Repo, it is not sufficiently resolved what is what. For that, a page (or two) in a Supplementary Methods File would be most suitable; and you should refer to this description when introducing the Dataverse Repo html address in the data availability statement. I suggest to use your Dataset list that you started off in response A3, correct for the shortcomings for the n-gram dataset as described below, and include descriptions for the missing 6 datasets (as you describe only 9 of 15 provided datasets).

Please refer for each dataset to the Figure where the data was used (if applicable), this makes it a lot easier for the reader to follow and identify relevant datasets. Please also add a dataset that was used for the wordcloud in Fig. 2A, as these word frequencies are currently missing. Please also include your word occurrence characterization from the rebuttal letter A3 (as a separate section) into the Supplementary Methods File that you could refer to in Fig. 2.

We have added a detailed description of the datasets to the repository under the name "Dataset_description.txt" and referenced it in the data availability statement. Additionally, each file in the repository is now annotated and linked to the corresponding figures. We opted not to include file naming information in the publication supplement, as we consider the repository a more dynamic and update-friendly format.

The word cloud is based on corpus-wide term frequencies, which are available in the chronic_thromboembolic_pulmonary_hypertension_TF_IDF_Drugs.csv file. Hence, a separate file for this data is unnecessary. This has been clarified in the Dataset_description.txt, in the caption of Figure 2 (now Fig. 3), and the file annotation of chronic_thromboembolic_pulmonary_hypertension_TF_IDF_Drugs.csv in the repo.

We have also created a literature corpora graphical characterization figure (Figure S1A [chronic thromboembolic pulmonary hypertension corpus] and Figure S1B [venous thrombosis corpus]) and a literature corpora statistical characterization table (Table S1), and added them to the supplement. They are referenced at the first point each literature corpus is mentioned in text (i.e. "The specific query for this study was "(chronic thromboembolic pulmonary hypertension) AND (english[Language])". Entrez provided 3,624 publication abstracts, of which 3,614 were accessible (Figure S1A and Table S1; retrieval date: July 11, 2024).")

My Q3 - n-gram analysis and Fig. 3

Please comment in the caption of Fig. 3 that you only show the "X" n-grams with highest frequency, and that these happen to be only 2-grams, but that there are some 3-grams with frequency >6. The files belonging to the n-gram analysis are not named such that one can make the link, e.g., "Disease_names_counts_ALL_clinical_features.csv" does not contain any hint on ngrams. Please alter the meta-description in the Dataverse, if possible, but at least the description of that dataset in the dataset list that I mentioned above. In addition, it appears that your results are truncated at a frequency of 6. Please describe this in the n-gram Methods part if you did this on purpose, and definitely also when describing the dataset.

A statement regarding the number of n-grams being predominantly 2-grams has been added to the Results section, and we have added to the caption of Figure 3 (now Fig. 4) that the top-20 n-grams including a clinical feature are shown, where only n-grams of n=[2,3,4,5] with frequency > 5 (to minimize noise) were considered. We further updated the text to clarify only n-grams of 2-5 and frequency > 5 were used to generate disease terms (Methods: "Clinical Features to Disease Terms" and Fig. 3 caption [now Fig. 4]).

We have furthermore added similar statements to the Dataverse file descriptions.

My Q7 - Word2Vec-based similarity analysis and Fig. 4

Please add the following information into the caption of Fig. 4: the numbers of drugs with direct CTEPH association, the numbers of drugs with indirect association and positive or negative thrombotic effects, and mention that you only show the Top X (?) results. These characterizations are very valuable for the reader to get the full output data picture from your tool and truthfully reveals the fact that the list of potential drugs you obtain is quite long. However, as it is now, this remains highly hidden as it can only be derived by processing some file from your Dataverse Repo. However, it should be very easy for you to derive and add.

We agree that this is useful information to understand how the top-ranked drug compounds stand out from the rest of the directly or indirectly associated drugs that remain in the reduced embedding space. Therefore, in the Results and Fig. 4 (now Fig. 5) caption we now break it down stepwise: the total number of drugs in the reduced embedding space, the number of these drugs that are directly vs. indirectly associated to CTEPH, and the number of these drugs that are labeled as anti- or pro-thrombotic. We also mention in the Fig. 4 (now Fig. 5) caption that we show the top 10 (directly associated) and top 6 (indirectly associated, per group: anti- or pro-thrombotic) drugs in the figure.

My Q2 – Alternative methods PrimeKG and Dreamwalk

Great that you checked these approaches with CTEPH! Actually, your description in the rebuttal letter is much more convincing than your extremely shortened formulation in the discussion. I think they would be very valuable for the readers. Please include them into a Supplementary Methods section, and refer to this description in the discussion (instead of the link to the Dataverse that is a _lot_ less instructive without the interpreting guidance here).

We have added a new section in the Supplementary Information called "Comparison with Alternative Methods: PrimeKG and DREAMwalk" where we include our information from the previous rebuttal letter, discussing installation, data exploration, and limitations encountered in

their applicability to CTEPH. This Supplementary Information section is now referenced in the Discussion section. We also moved our referral to the PrimeKG output in the Dataverse from the main text to the Supplementary section, which now includes the following statement: “The full PrimeKG output for nodes connected to CTEPH is available in the Dataverse repository.”

Minor comment: The word “PubDigest” appears only exactly once (except for the running title), in line 353. I recommend to introduce somewhere explicitly what you mean with it.

We now introduce our tool as PubDigest and reference it as PubDigest throughout the manuscript.
